# Health impact assessment to predict the impact of tobacco price increases on COPD burden in Italy, England and Sweden

**DOI:** 10.1038/s41598-021-81876-3

**Published:** 2021-01-27

**Authors:** Elaine Fuertes, Alessandro Marcon, Laura Potts, Giancarlo Pesce, Stefan K. Lhachimi, Virjal Jani, Lucia Calciano, Alex Adamson, Jennifer K. Quint, Debbie Jarvis, Christer Janson, Simone Accordini, Cosetta Minelli

**Affiliations:** 1 National Heart and Lung Institute, Emmanuel Kaye Building, Imperial College London, 1B Manresa Road, London, SW3 6LR UK; 2 Unit of Epidemiology and Medical Statistics, Department of Diagnostics and Public Health, University of Verona, Verona, Italy; 3 Institute of Psychiatry, Psychology and Neuroscience, King’s College London, London, UK; 4 Sorbonne Université, INSERM UMR-S 1136, Epidemiology of Allergic and Respiratory Diseases (EPAR), Pierre Louis Institute of Epidemiology and Public Health (IPLESP), Saint-Antoine Medical School, Paris, France; 5 Health Sciences Bremen, Institute for Public Health and Nursing, University of Bremen, Bremen, Germany; 6 MRC Centre for Environment and Health, Imperial College London, London, UK; 7 Department of Medical Sciences: Respiratory, Allergy and Sleep Research, Uppsala University, Uppsala, Sweden

**Keywords:** Chronic obstructive pulmonary disease, Epidemiology, Risk factors, Public health

## Abstract

Raising tobacco prices effectively reduces smoking, the main risk factor for chronic obstructive pulmonary disease (COPD). Using the Health Impact Assessment tool “DYNAMO-HIA”, this study quantified the reduction in COPD burden that would occur in Italy, England and Sweden over 40 years if tobacco prices were increased by 5%, 10% and 20% over current local prices, with larger increases considered in secondary analyses. A dynamic Markov-based multi-state simulation modelling approach estimated the effect of changes in smoking prevalence states and probabilities of transitioning between smoking states on future smoking prevalence, COPD burden and life expectancy in each country. Data inputs included demographics, smoking prevalences and behaviour and COPD burden from national data resources, large observational cohorts and datasets within DYNAMO-HIA. In the 20% price increase scenario, the cumulative number of COPD incident cases saved over 40 years was 479,059 and 479,302 in Italy and England (populous countries with higher smoking prevalences) and 83,694 in Sweden (smaller country with lower smoking prevalence). Gains in overall life expectancy ranged from 0.25 to 0.45 years for a 20 year-old. Increasing tobacco prices would reduce COPD burden and increase life expectancy through smoking behavior changes, with modest but important public health benefits observed in all three countries.

## Introduction

Tobacco consumption remains the largest preventable risk factor for death and disease in the European Union, with 50% of smokers dying prematurely and many more suffering from smoking-related diseases^1^. Although estimated population attributable risks vary widely between studies^2^, there is overwhelming evidence that smoking is the most important risk factor for chronic obstructive pulmonary disease (COPD), a condition responsible for 3.2 million deaths and 2.6% of disability-adjusted life years (in 2015) worldwide^3^.

Various policies have been implemented to reduce smoking initiation and encourage cessation, at both national and the European Union level (e.g. 2014 European Tobacco Products Directive)^4^. However, smoking initiation rates continue to increase among 11–15 year-olds in most of Europe, especially in the West, and remain high among 16–20 year-olds in East, South and West Europe^5^. As over half of smokers begin to smoke before the age of 18 years^1^, policies that discourage smoking initiation (especially in adolescence)^6^ and encourage cessation at all ages are likely to be most beneficial^7^. In particular, male early adolescence represents an important window of opportunity for preventive interventions that may benefit several generations, as tobacco smoking before 15 years of age in men may have a negative impact on respiratory health in future offspring^8^. Increasing tobacco prices may also help break the chain of tobacco supply to the youngest age groups experimenting with smoking, who tend to obtain cigarettes from their peers^9,10^. Overall, raising the price of tobacco is considered to be an effective policy to reduce cigarette smoking^11^, and is likely to be accepted by most of the population, as suggested by the World Health Organization’s MPOWER initiative (the “R” representing “Raise taxes on tobacco”)^12^.

Health Impact Assessment (HIA) is a tool to derive quantitative predictions of the influence of a successfully implemented intervention on health and has been used to assess the expected impact of increasing the price of tobacco on future COPD burden in single-country analyses in the European Union^13–15^. These previous analyses suggest that raising tobacco prices would reduce future COPD burden in different European countries, but direct comparisons of their results are hampered by substantial differences in methodology and interventions tested.

As part of the “Ageing Lungs in European Cohorts” (ALEC) consortium (https://www.alecstudy.org), we quantified the reduction in COPD burden, through a reduction in smoking prevalence, that would occur in Italy, England and Sweden over a 40-year time frame if tobacco prices were increased by 5%, 10% and 20% over current local prices using the standardized, flexible and publicly available tool “DYNAMO-HIA” (https://www.dynamo-hia.eu^16^), which has been used for similar exercises^14,17^. These price increases were selected as they represent measures that could be relatively easily and quickly implemented by governments, but larger increases (30%, 40% and 50%) were considered in secondary analyses. We also estimated the gain in life expectancy that could be expected through a reduction in overall mortality associated with a reduction in smoking. We aimed to identify whether there were differences between countries in the effectiveness of price interventions in reducing the national COPD burden. We chose Italy, England and Sweden as our three countries of interest because of their substantial differences in tobacco prices, ranging from 4.9 euros in Italy to 10.3 euros in England for a pack of 20 cigarettes^18^; in terms of purchasing power parity, the price of the most sold brand of cigarettes in 2018 ranged from 7.35 international dollars in Sweden to 13.58 international dollars in England^19–21^. These countries also vary in smoking prevalences (ranging from 10.6% in Sweden to 17.5% in Italy in 2018) and COPD burden (prevalence ranging from 0.9% in England to 1.3% in Sweden in 2018). Populations from these countries also have markedly different age distributions, which is likely to affect the local effectiveness of tobacco price interventions.

## Results

### Effect of interventions on smoking behaviour

Current, former and never smoking prevalences per age, country and intervention at baseline (2018) are presented in Supplementary Figs. S1-S3, and the smoking initiation, cessation and restart rates per age and region (south/north) used throughout the simulation in Supplementary Fig. S4–S6. Smoking prevalences in the reference and intervention scenarios in 2018 and at the end of the simulation (2058) are presented in Table 1 (entire simulation for the overall population, males and females presented in Supplementary Figs. S7, S8 and S9, respectively).

**Table 1:** Prevalence of current, former and never smoking (expressed in %) at baseline (2018) and at the end of the 40-year simulation (2058).

Smoking status	Scenario	Italy		England		Sweden	
		2018	2058	2018	2058	2018	2058
Current	Reference	17.5	15.3	16.4	9.0	10.6	8.2
	5% tobacco price increase	15.4	14.1	14.3	8.2	8.4	7.4
	10% tobacco price increase	13.6	12.9	12.5	7.4	6.6	6.7
	20% tobacco price increase	10.5	10.8	9.2	6.1	3.3	5.4
Former	Reference	20.4	16.4	19.9	15.3	21.6	12.2
	5% tobacco price increase	20.9	15.9	20.4	14.6	22.2	11.5
	10% tobacco price increase	21.4	15.4	20.9	14.0	22.7	10.8
	20% tobacco price increase	22.0	14.5	21.6	12.8	23.2	9.5
Never	Reference	62.1	68.3	63.7	75.7	67.8	79.6
	5% tobacco price increase	63.7	70.0	65.3	77.2	69.4	81.1
	10% tobacco price increase	65.1	71.6	66.6	78.5	70.8	82.5
	20% tobacco price increase	67.5	74.7	69.2	81.1	73.4	85.1

Results reported per country and scenario.

There was a reduction in overall current smoking prevalence of 4.5%, 2.9% and 2.8% between the reference scenario and 20% tobacco price increase scenario for Italy, England and Sweden, respectively, by the end of the 40-year simulation in 2058. In all countries, tobacco price increases led to higher prevalences of never smokers and the prevalence of former smokers was lower in the intervention scenarios than in the reference scenario by 2058 (Supplementary Fig. S7).

### Effect of interventions on COPD prevalence and incidence

**Figure 1:**
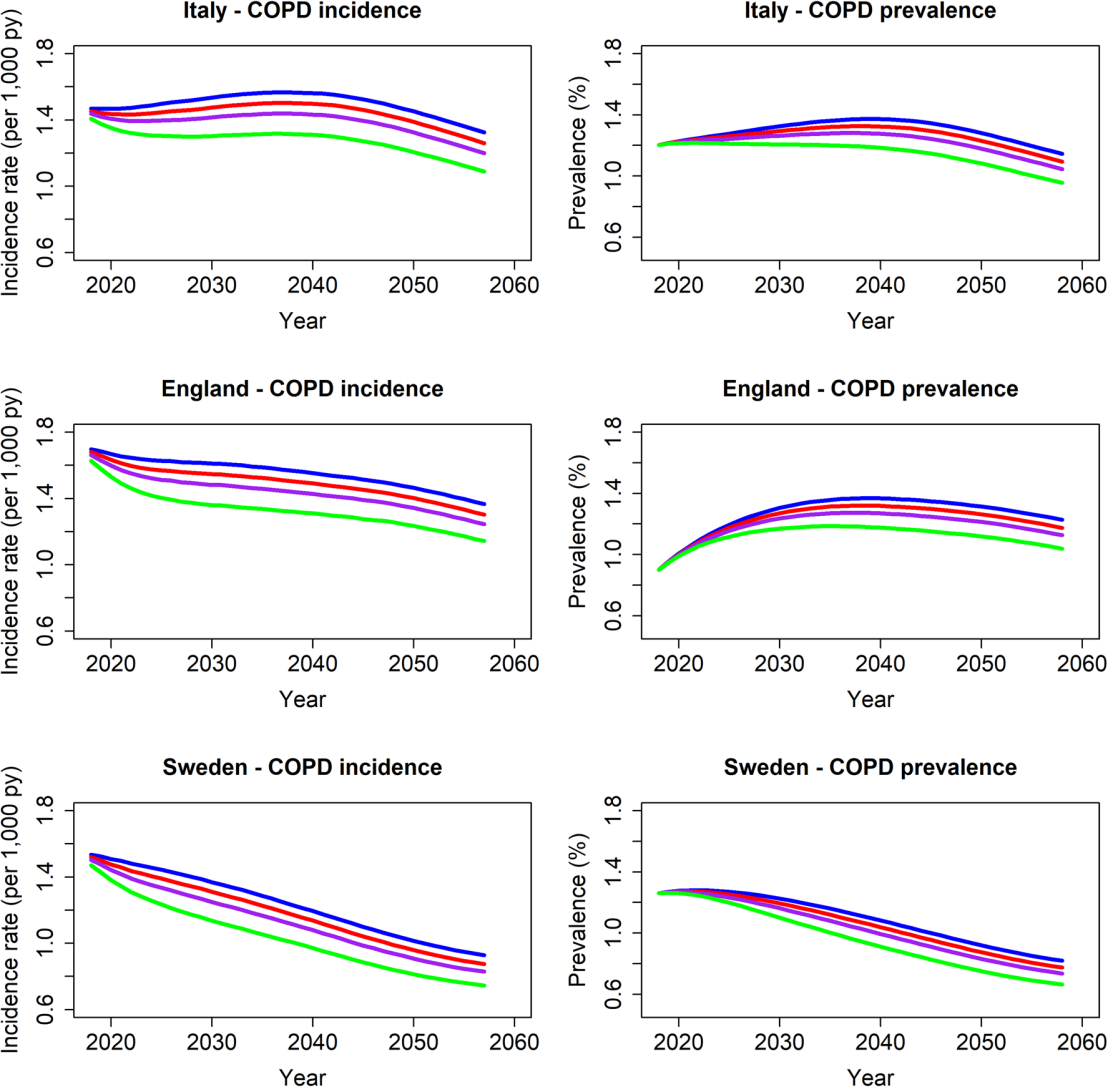
Projected COPD incidence (cases/1000 person-years) and COPD prevalence (%) over a 40-year time period in the various scenarios (blue: reference scenario; red, purple and green: 5%, 10% and 20% tobacco price increase, respectively). Figure created using the statistical program R (version 3.6.2, https://www.R-project.org).

At the start of the simulation (2018) in the reference scenario, COPD prevalence was lowest in England (0.9%) and highest in Sweden (1.3%), whereas COPD incidence rates were lowest in Italy (1.5 per 1000 person-years) and highest in England (1.7 per 1000 person-years). Country-specific COPD prevalence and incidence rates over the entire simulation are presented in (Fig. 1).

COPD prevalences 10, 20 and 40 years after the start of the simulation in each scenario are presented per country in Table 2 for the overall population (sex-specific results in Supplementary Table S1). Compared to the reference scenario, reductions in COPD prevalence are modest for all intervention scenarios. After 10-year, reductions ranged from 0.03% in the 5% tobacco price increase scenario in all countries to 0.12% in England, 0.11% in Sweden and 0.10% in Italy in the 20% price increase scenario; reductions after 20 and 40 years are slightly larger and more similar across countries, ranging from 0.16% to 0.19% in the 20% price increase scenario.

**Table 2:** COPD burden (prevalence and incidence) 10 (2028), 20 (2038) and 40 (2058) years from the implementation of the intervention.

Health measure	Scenario	Italy			England			Sweden		
		2028	2038	2058	2028	2038	2058	2028	2038	2058
COPD prevalence (%)	Reference	1.31	1.37	1.15	1.27	1.37	1.23	1.25	1.11	0.82
	5% tobacco price increase	1.28	1.33	1.09	1.24	1.32	1.18	1.22	1.07	0.78
	10% tobacco price increase	1.26	1.28	1.05	1.21	1.27	1.13	1.19	1.03	0.74
	20% tobacco price increase	1.21	1.19	0.96	1.15	1.18	1.04	1.14	0.95	0.66
COPD incidence (cases/1,000 person-years)^a^	Reference	1.52	1.57	1.32	1.62	1.57	1.37	1.40	1.23	0.93
	5% tobacco price increase	1.46	1.50	1.26	1.56	1.50	1.30	1.34	1.17	0.87
	10% tobacco price increase	1.41	1.44	1.20	1.49	1.44	1.24	1.28	1.11	0.83
	20% tobacco price increase	1.30	1.32	1.09	1.37	1.32	1.14	1.17	1.01	0.75

Results reported per country and scenario.

^a^The COPD incidence value after 39 years (2057) instead of 40 years (2058) is given as DYNAMO does not calculate COPD incidence rates for the last year of the simulation.

COPD incidence rates 10, 20 and 39 years after the start of the simulation in each scenario are presented per country in Table 2 for the overall population (sex-specific results in Supplementary Table S2). Compared to the reference scenario, overall COPD incidence rates are between 0.06 and 0.07 cases/1000 person-years lower throughout the entire simulation for the 5% tobacco price increase scenario in all countries. This reduction is slightly larger in the 10% and 20% tobacco price increase scenarios (reductions of 0.10–0.13 cases/1000 person-years and 0.18–0.25 cases/1000 person-years, respectively).

The total cumulative number of COPD incident cases saved over the entire 40-year simulation for the 5%, 10% and 20% price increase interventions compared to the reference scenario were respectively: 124,364, 247,364 and 479,059 in Italy; 125,712, 248,707 and 479,302 in England; 22,070, 43,654 and 83,694 in Sweden.

### Effect of interventions on life expectancy

There are gains in years of overall life expectancy, COPD-free life expectancy and COPD-disability-adjusted life expectancy in the intervention scenarios compared to the reference scenario for both sexes (Table 3). Those with more time to benefit from the intervention have greater improvements. For example, on average, Italian males aged 20 years at the start of the simulation (2018) who have a life expectancy of 80.3 years in the reference scenario, would gain 0.10, 0.22 and 0.43 years of overall life expectancy in the 5%, 10% and 20% tobacco price increase intervention scenarios, respectively. The corresponding gains for Italian males aged 60 years in 2018 are only 0.04, 0.09 and 0.16 years, respectively. In terms of gains in COPD-free and COPD-disability adjusted life expectancy, values ranged from 0.13 to 0.57 years and 0.08 to 0.38 years, respectively, for males aged 20 years in 2018 across the three countries. Overall, gains in life-expectancy are similar across countries.

**Table 3:** Absolute gain in overall, COPD -free, and COPD-disability-adjusted cohort life-expectancy (expressed in years) of a 20, 40 and 60 year-old male and female at baseline (2018), as compared to the reference scenario.

	Sex	Scenario	Italy			England			Sweden		
			Gain in years of life expectancy			Gain in years of life expectancy			Gain in years of life expectancy		
			Overall	COPD-free	COPD-disability-adjusted	Overall	COPD-free	COPD-disability-adjusted	Overall	COPD-free	COPD-disability-adjusted
20 year- old	Males	5% tobacco price increase	0.10	0.13	0.08	0.14	0.17	0.11	0.11	0.13	0.09
		10% tobacco price increase	0.22	0.29	0.19	0.24	0.31	0.21	0.20	0.26	0.17
		20% tobacco price increase	0.43	0.55	0.36	0.45	0.57	0.38	0.43	0.54	0.36
	Females	5% tobacco price increase	0.10	0.15	0.09	0.08	0.12	0.07	0.06	0.10	0.06
		10% tobacco price increase	0.17	0.24	0.14	0.16	0.24	0.14	0.11	0.16	0.09
		20% tobacco price increase	0.33	0.46	0.27	0.31	0.45	0.26	0.25	0.38	0.22
40 year- old	Males	5% tobacco price increase	0.08	0.10	0.06	0.09	0.12	0.07	0.09	0.12	0.08
		10% tobacco price increase	0.15	0.21	0.13	0.19	0.25	0.16	0.20	0.28	0.18
		20% tobacco price increase	0.33	0.44	0.28	0.40	0.54	0.35	0.36	0.50	0.32
	Females	5% tobacco price increase	0.05	0.07	0.04	0.07	0.11	0.06	0.06	0.10	0.06
		10% tobacco price increase	0.12	0.17	0.10	0.14	0.22	0.13	0.11	0.18	0.10
		20% tobacco price increase	0.25	0.36	0.21	0.29	0.45	0.26	0.23	0.37	0.21
60 year- old	Males	5% tobacco price increase	0.04	0.06	0.04	0.04	0.06	0.04	0.03	0.05	0.03
		10% tobacco price increase	0.09	0.12	0.07	0.09	0.13	0.08	0.07	0.11	0.06
		20% tobacco price increase	0.16	0.22	0.14	0.23	0.31	0.19	0.16	0.23	0.14
	Females	5% tobacco price increase	0.06	0.08	0.05	0.06	0.09	0.05	0.04	0.06	0.03
		10% tobacco price increase	0.11	0.15	0.09	0.11	0.17	0.09	0.06	0.10	0.05
		20% tobacco price increase	0.19	0.27	0.15	0.21	0.32	0.17	0.12	0.20	0.11

Results reported per country and intervention scenario.

### Price elasticity sensitivity analysis

With the same price elasticity coefficient of − 0.5 for all ages, there would be a slightly smaller reduction in current smoking prevalences in the intervention scenarios compared to the reference scenario. For example, after 40 years, the reduction in the overall prevalence of current smokers in the 20% tobacco price increase scenario, compared to the reference scenario, would be 4.5%, 2.9% and 2.8% in the main analysis for Italy, England and Sweden, respectively, while the corresponding values are 2.8%, 1.8% and 1.7% when all price elasticities are set to − 0.5. The health benefits (i.e. lower COPD prevalence, fewer COPD incident cases and gains in life expectancy) are slightly attenuated in this sensitivity analysis (Supplementary Table S3).

### Price increase secondary analysis

As expected, intervention scenarios of higher tobacco price increases (i.e. 30%, 40% and 50%) evaluated as secondary analyses yielded greater gains for both COPD prevalence and incidence throughout the simulations as compared to the main analysis (Table S4). Similarly, there were greater gains in years of overall life expectancy, COPD-free life expectancy and COPD-disability-adjusted life expectancy for both sexes in these secondary analyses as compared to the main analysis (Table S5).

## Discussion

This study shows that increasing the price of tobacco would reduce current smoking prevalences and associated COPD burden, with modest but important public health benefits observed within 40 years. For example, although the COPD incidence rate in the 20% price increase scenario in England would be only 0.23 cases/1000 person-years lower than in the reference scenario in 2058, the cumulative number of COPD incidence cases saved from 2018 to 2058 would be 479,302. By using the same methodology for the HIA analyses in three European countries, this study also demonstrates that baseline smoking prevalences and projected changes in the population structure of a country will influence the effectiveness of national tobacco reduction policies.

Trends in overall smoking prevalences in the reference and intervention scenarios differed slightly by country, which likely reflects demographic changes in the populations (Supplementary Fig. S10). For example, in Italy, population projections indicate a fall in the percentage of the population that is middle-aged (45–64 years of age) between 2018 and 2058 (29% and 23% in 2018 and 2058, respectively). This age group has the highest smoking prevalence on which the intervention will act.

Projected changes in population structure may also explain differences in the trends of COPD incidence rates over time. In Italy, despite reductions in current smoking prevalences, a slight increase or plateauing in the COPD incidence rates are observed for the first 20 years of the simulation, before a steady decrease becomes apparent. This trend likely occurs because the proportion of older individuals in the population at risk of COPD increases during that time. In comparison, the age-distribution of the population in England and Sweden is expected to remain more stable and the decreases in COPD incidence are apparent for all scenarios throughout the entire simulation.

For the minimum price increase of 5%, 124,364, 125,712 and 22,070 excess COPD cases could be prevented over a 40-year period in Italy, England and Sweden, respectively, compared to the reference scenario. According to a recent systematic review^22^, the mean direct annual cost per COPD patient due to disease management in nine European countries is approximately 5700 euros. Using this cost value and assuming each patient lives 10 years with the disease, more than 7 billion euros could be saved in Italy and England, and over 1.2 billion euros in Sweden, if the COPD cases under the minimum price increase scenario were prevented over the 40-year study period. These cost savings are certainly underestimates as indirect costs, such as loss in work productivity and early retirement costs, are not considered. When including the mean annual cost due to work productivity losses in Italy (397 euros) and Sweden (998 euros) (not reported for the United Kingdom)^22^ to this annual cost of disease management, cost savings would rise to over 7.5 billion and over 1.4 billion euros in these two countries, respectively. Although these figures are only indicative, they provide a picture of the substantial cost savings that could be achieved with a relatively simple public health intervention of a one-time increase in tobacco price.

Our analysis is largely based on existing data provided within the DYNAMO-HIA tool. However, we added up-to-date estimates for population size, projected newborns, and importantly, smoking state prevalences and the probabilities of transitioning between smoking states, using newly available estimates derived from six large multicentre observational studies participating in the ALEC consortium. Of particular interest is the use of European region-specific probabilities of smoking initiation and cessation, which better reflect known geographical differences in smoking patterns in Europe^5,23^. We also incorporated data specifically on young individuals (as of 11 years) to model potential benefits for the entire population who may be affected by tobacco price increases.

Some limitations should be noted. First, any defined intervention scenario remains a limited reflection of real-world settings. Previous work has assumed that only parts of the population would be affected by an increase in tobacco price (e.g. only never smokers < 21 years and former smokers ≥ 21 years^14^, or only initiation rates for ≤ 18 years and cessation and restart rates for > 25 years)^15^. In the current analysis, we allowed the whole population and all smoking states and probabilities of transitioning between these states to be affected. We aimed to capture the different behaviours of a population by using three different price elasticities; younger populations were set to be more responsive to increases in tobacco prices because of a presumably lower disposable income. We conducted a sensitivity analysis in which a lower price elasticity − 0.5) was used for all ages, as done in a recent analysis in the United Kingdom^13^, which resulted in slightly smaller reductions in current smoking prevalences and corresponding health benefits in the intervention scenarios compared to the reference scenario. Previous studies in Europe have also used even lower price elasticities (e.g. ranging from − 0.1 to − 0.3 depending on age-group)^24^. Individual responses to changing prices are likely to vary by other sociodemographic characteristics than age (e.g. income, socioeconomic status, sex)^25^, which were not captured in this analysis. Finally, although we assumed that the price elasticity of tobacco is the same throughout all three countries, there is evidence to suggest that it may be higher in areas between countries with a large price differential, possibly reflecting increased large-scale illicit trade and cross-border purchasing^26^. However, the true extent of the effect of these factors on overall smoking prevalence continues to be debated^27,28^.

We also assumed that individuals of the same age group would be similarly affected by a percentage price increase in all countries despite differences in earnings across countries. Tobacco is most affordable in Italy and Sweden and least affordable in England. The 2020 weighted average price of a pack of 20 cigarettes in euros was 4.9 in Italy, 5.9 in Sweden and 10.3 in England^18^. After adjusting for purchasing power parity, the corresponding prices for the most sold brand of cigarettes in 2018 were 7.35, 7.49 and 13.58 international dollars in Sweden, Italy and England, respectively^19–21^. Moreover, one could speculate that age groups of the population with more limited disposable income, such as adolescents, living in countries where the price of tobacco is already high, may be more likely to alter their behavior even when small price increases are applied compared to those with more disposable income. It should also be noted that we did not evaluate how changes in income within a country over the 40-year timeframe considered may change tobacco consumption and subsequently COPD burden. For example, economic recessions have been shown to be associated with significant decreases in the prevalence of smoking^29^.

Although some previous analyses have modelled the effect of large (50%^30^) or annual (5% per year^13^) tobacco price increases, we chose to model the effects of a one-time price increase of 5%, 10% and 20% in our main analysis, as measures that could be relatively easily and quickly implemented by governments. However, countries such as Italy where tobacco is more affordable could more easily consider price increases substantially larger than the 20% modelled as the maximum scenario in the main analysis in order to bring the price of tobacco closer to that of countries were tobacco is less affordable, such as in the United Kingdom, which would result in larger public health benefits. This suggests that the health benefits simulated in the main analysis may be conservative estimates of what could be feasibly achieved, and higher price increases may be warranted. In secondary analyses in which scenarios of larger price increases of 30%, 40% and 50% were evaluated, greater health benefits were clearly apparent as would be expected. A strategy consisting of yearly cumulative price increases, instead of a single one-time price increase, would also undoubtedly yield greater benefits, which suggests our main results are likely conservative estimates of the potential benefits.

The DYNAMO-HIA model does not account for any other policy-related changes that may occur throughout the timeframe of the simulation. For example, the reference scenario assumed that the price of tobacco remained constant, which does not truly reflect reality. In the United Kingdom, the price of all tobacco products increases 2% above Retail Price Index inflation every year^31^. A recent analysis in the United Kingdom evaluated the impact of a yearly 5% price increase of tobacco above inflation, assuming a price elasticity value of − 0.5. This study found that 3.3% of incident COPD cases could be avoided in one year, after allowing the simulation to run 20-years, compared to the reference scenario in which the price of tobacco followed the regulatory 2% yearly increase^13^. We attempted to replicate this result as closely as possible following our methods (one-time 5% tobacco price increase at baseline, price elasticity value of − 0.5). Although the absolute number of COPD incident cases avoided is larger in our study, we obtained a similar percent reduction in incident COPD cases (4.0%) in one year after running the simulation 20 years compared to the reference scenario in which no price increase was applied. Differences between these results are likely due to the use of different data sources (e.g. COPD prevalence data was obtained from Public Health England and the Office for National Statistics in the previous analysis^13^, whereas the current analyses used country-specific data incorporated in DYNAMO-HIA) and price interventions, among other factors. The countries included in our analysis also differ in terms of their specific existing tobacco control policies, although all completely (or moderately) meet many of the recommended WHO MPOWER measures to reduce the demand for tobacco (cessation programs in place, monitoring of smoking prevalence, taxation, advertising bans, use of health warnings on packaging, etc.)^19–21^.

Our model was unable to assess how tobacco price increases might change smoking intensity and, the use of alternative products, such as electronic cigarettes, pipe tobacco or smokeless tobacco products, or the illicit tobacco trade. A rise in tobacco prices might well increase the use of electronic cigarettes and smokeless tobacco products, some of which have been suggested to affect health outcomes^32,33^, although there is currently no consistent evidence for an effect on COPD development. Future work should investigate these unknowns, as 20% of respondents to the European Eurobarometer survey (in 2017) reported at least trying electronic cigarettes^1^. Equalising taxation levels of all tobacco products may be an effective strategy to limit any type of smoking initiation, as advocated by the Framework Convention on Tobacco Control^34^. Finally, population structure changes attributable to immigration are not considered within DYNAMO-HIA.

In conclusion, this study demonstrates to policy makers that feasible increases in the price of tobacco which could easily and quickly be implemented would reduce COPD burden, with modest but still important public health benefits observed within 40 years. Baseline smoking prevalences and projected changes in population structure of a country are important factors influencing the effectiveness of the intervention and need to be considered when comparing the potential impact of tobacco price increases on public health outcomes in different countries. It is also worthwhile noting that additional benefits of increasing the price of tobacco and a subsequent decrease in smoking prevalence should be expected. These benefits include lower passive smoking exposures, higher tobacco taxation revenues that could be used for improving health care^35^ and supporting tobacco cessation initiatives/treatments^12^, a lower environmental burden of cigarettes^36^, and importantly, a reduction in the morbidity and mortality from many other smoking-related diseases.

## Methods

### The DYNAMO-HIA model

We used the publicly available and flexible DYNAMO-HIA tool to conduct the present HIA analysis (https://www.dynamo-hia.eu, version 2.0.8), which has been used to model health impacts following changes in various risk factors^14,15,17,37–42^. Our analysis consists of a dynamic Markov-based multi-state simulation modeling of the age- and sex-specific probabilities of individuals staying in or moving to a particular risk factor state in different intervention scenarios defined by the user, compared to a reference scenario. A detailed description of this tool has been published^16,43^ and is detailed online on the DYNAMO-HIA website (https://www.dynamo-hia.eu). Summary information of the model specifications is provided in the Supplemental Material. We simulated the effect of changes in smoking prevalence states (current/ex/never smoker) and rates of transitioning between these states (smoking initiation, cessation and restart) due to increases in local tobacco prices on future smoking prevalence, COPD prevalence, COPD incidence and gains in life expectancy in Italy, England and Sweden.

### Data sources

The model input parameters consisted of data on demographics, smoking prevalences and behaviour as well as COPD burden. Except for population size and projected number of newborns, all data were stratified by sex and year of age for the entire lifespan (0–95 years of age). When data were only available per age group (e.g. per five years rather than per single year), the same value was applied to all ages in that group. The relative risk of smoking on COPD was provided by sex and age group (ranged from 8.13 to 19.83) and the relative risk of smoking on total mortality by sex (2.07 for males and 1.74 for females).

The data used and its sources are summarized in Supplementary Table S6. Most datasets used are integrated within the DYNAMO-HIA tool^16^ (further details available: https://www.dynamo-hia.eu/en/documents-and-publications) with three notable exceptions. First, we used updated information on population size and projected number of newborns obtained from national sources. Second, smoking prevalences were based on recent data from large country-specific population-based observational studies or representative national surveys, as described in the Supplementary File and summarised in Supplementary Table S6. Third, the probabilities of transitioning between smoking states (initiation/cessation/restart) were calculated per age, sex and region in Europe (Italy considered “South” and England and Sweden both considered “North”, corresponding to the United Nations geoscheme and tobacco epidemiology^44^) using pooled data from six large multicentre observational studies participating in the ALEC consortium, as previously described^5,23^.

### Tobacco price increase scenarios

Four scenarios were evaluated: the price of tobacco was left unchanged (“reference scenario”, sometimes labelled as “business-as-usual” scenario) or was increased once by 5%, 10% and 20% above local prices at the beginning of the simulation period (“intervention scenarios”).

The purpose of the reference scenario is to determine future smoking prevalence and burden of COPD if no intervention takes place. Accordingly, no changes were applied to the smoking state prevalences or probabilities of transitioning between smoking states.

In the three intervention scenarios, the price elasticity of demand for tobacco (i.e. the percentage change in consumption resulting from a 1% increase in price) was used to calculate the impact of increasing tobacco prices on smoking prevalence and the probabilities of transitioning between smoking states. In line with previous work^13,45^, we used − 0.5 as the coefficient of price elasticity for adults (≥ 30 years of age). For younger populations who are more sensitive to price changes, we assumed a higher coefficient of price elasticity of − 0.75 for 19–29 year-olds and − 1.5 for < 19 year-olds^46,47^. In a sensitivity analysis, we repeated the analysis using − 0.5 as the coefficient of price elasticity for all ages, as done recently in an analysis in the United Kingdom^13^.

### Statistical analyses

All possible smoking states were categorized as never, former and current. Additionally, we made the following assumptions:

- Smoking could only begin as of 11 years of age, since it has been reported that uptake of smoking at younger ages is unlikely^5^;
- Smoking cessation was only possible as of 16 years of age^23^. Restarting smoking was only possible as of 22 years of age. These cut-offs were selected because of the difficulty in generating precise estimates of cessation and restart rates at younger ages because of data sparseness;
- As summarized in Table 4, an increase in tobacco price would affect both the prevalence of never smokers (as fewer people start smoking) and former smokers (as current smokers quit) by the same percentage. Consequently, the prevalence of current smokers was calculated as the complement to 100% of the prevalence of never and former smokers. We also assumed that the rates of initiation, cessation and restart would be affected by the same percentage. All changes were assumed to occur at the beginning of the simulation (i.e. there is no delay in the delivery of the intervention);
- As COPD onset is rarer before age 40 years, COPD prevalence was only considered as of this age^48^.

**Table 4:** Predicted changes in smoking prevalence and smoking state probabilities attributable to a one-time tobacco price increase.

		Age groups				
		11–15 years	16–18 years	19–21 years	22–29 years	> 30 years
Price elasticity coefficient^a^		− 1.5	− 1.5	− 0.75	− 0.75	− 0.5
Smoking prevalence	Current	↓ of (100 – never)	↓of (100 − (never + former))	↓ of (100 − (never + former))	↓ of (100 − (never + former))	↓ of (100 − (never + former))
	Former	NA	↑ of (1.5)•(% price increase)	↑ of (0.75)•(% price increase)	↑ of (0.75)•(% price increase)	↑ of (0.5)•(% price increase)
	Never	↑ of (1.5)•(% price change)	↑ of (1.5)•(% price increase)	↑of (0.75)•(% price increase)	↑ of (0.75)•(% price increase)	↑ of (0.5)•(% price increase)
Smoking state probabilities	Initiation	↓ of (1.5)•(% price change)	↓ of (1.5)•(% price increase)	↓ of (0.75)•(% price increase)	↓ of (0.75)•(% price increase)	↓ of (0.5)•(% price increase)
	Cessation	NA	↑ of (1.5)•(% price increase)	↑ of (0.75)•(% price increase)	↑ of (0.75)•(% price increase)	↑ of (0.5)•(% price increase)
	Restart	NA	NA	NA	↓ of (0.75)•(% price increase)	↓ of (0.5)•(% price increase)

NA = no change possible because of constraints placed on age ranges (e.g. smoking cessation only possible ≥ 16 years of age).

^a^Defined as the percentage change in tobacco per 1% increase in tobacco price.

Using a simulated population for each year of age (from 0 to 95) and sex, we performed country-specific analyses over a 40-year time period (from 2018 to 2058). Given the long latency period of COPD, this long timeframe was required to allow changes in COPD prevalence due to changes in smoking patterns in younger age groups to be observed. All figures were created using the statistical program R (version 3.6.2)^49^ except Figure S10 which was created using the DYNAMO-HIA tool.

### Secondary analyses

We a priori chose to model the effects of a one-time price increase of 5%, 10% and 20% in our main analysis as measures that could be relatively easily and quickly implemented by governments. In a secondary post-hoc analysis, we estimated the health benefits that would be expected if larger tobacco price increases of 30%, 40% and 50% were used instead, which would be harder to implement by governments but would likely lead to larger reductions in smoking prevalence and corresponding COPD burden.

## Supplementary Information

Supplementary information.

## Data Availability

Nearly all data used are publicly available (data sources listed in Table S6). Only components of the smoking prevalence data as well as the smoking initiation, restart and cessation probabilities have not previously been published and are available to interested researchers from the corresponding author upon reasonable request.
