# Supplementary material for: Health impact assessment to predict the impact of tobacco price increases on COPD burden in Italy, England and Sweden
Source: Sci Rep. 2021 Jan 27;11:2311. doi: 10.1038/s41598-021-81876-3 (PMC7840977; doi:10.1038/s41598-021-81876-3)
Supplement: Supplementary file 1 — Supplementary information. [file 41598_2021_81876_MOESM1_ESM.pdf]

SUPPLEMENTAL MATERIAL

Health Impact Assessment to predict the impact of tobacco price increases on COPD burden in Italy, England and Sweden

Elaine Fuertes[1], Alessandro Marcon[2], Laura Potts[3], Giancarlo Pesce[4], Stefan K Lhachimi[5], Virjal Jani[1], Lucia Calciano[2], Alex Adamson[1], Jennifer K Quint[1], Debbie Jarvis[1,6], Christer Janson[7], Simone Accordini[2†], Cosetta Minelli[1†]

1- National Heart and Lung Institute, Imperial College London, London, United Kingdom
2- Unit of Epidemiology and Medical Statistics, Department of Diagnostics and Public Health, University of Verona, Verona, Italy
3- Institute of Psychiatry, Psychology & Neuroscience, King's College London, London, United Kingdom
4- Sorbonne Université, INSERM UMR-S 1136, Epidemiology of Allergic and Respiratory Diseases (EPAR), Pierre Louis Institute of Epidemiology and Public Health (IPLESP), Saint-Antoine Medical School, Paris, France
5- Health Sciences Bremen, Institute for Public Health and Nursing, University of Bremen, Bremen, Germany.
6- MRC-PHE Centre for Environment and Health, Imperial College London, London, United Kingdom
7- Department of Medical Sciences: Respiratory, Allergy and Sleep research, Uppsala University, Uppsala, Sweden

[†] Shared senior author

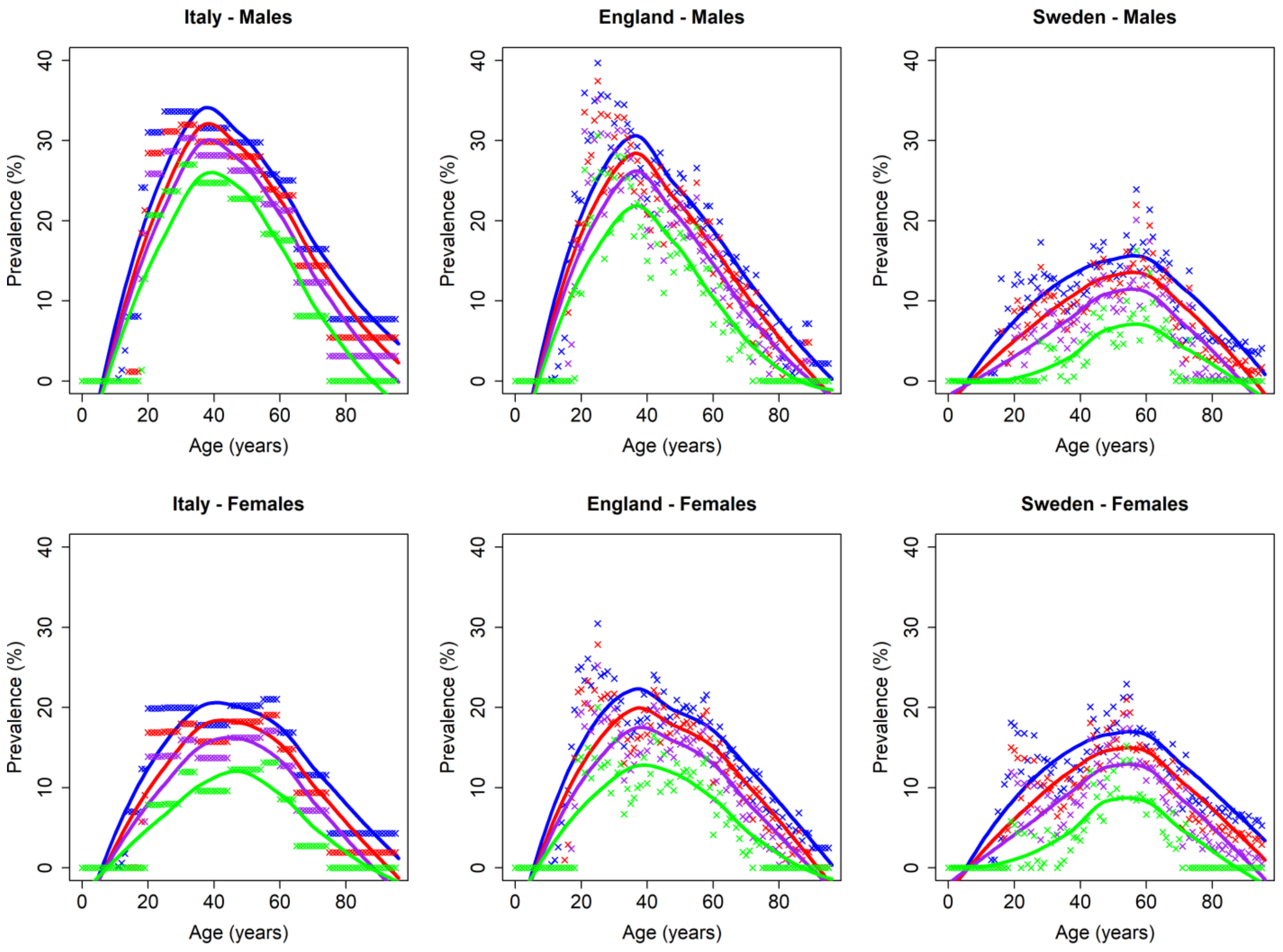

Figure S1: Baseline (2018) **current smoking** prevalences per age, country and intervention (blue: reference scenario; red, purple and green: 5%, 10% and 20% tobacco price increase, respectively). Locally weighted scatterplot smoothing (lowess) was used to create a smooth line through the points. Figure created using the statistical program R (version 3.6.2, www.R-project.org).

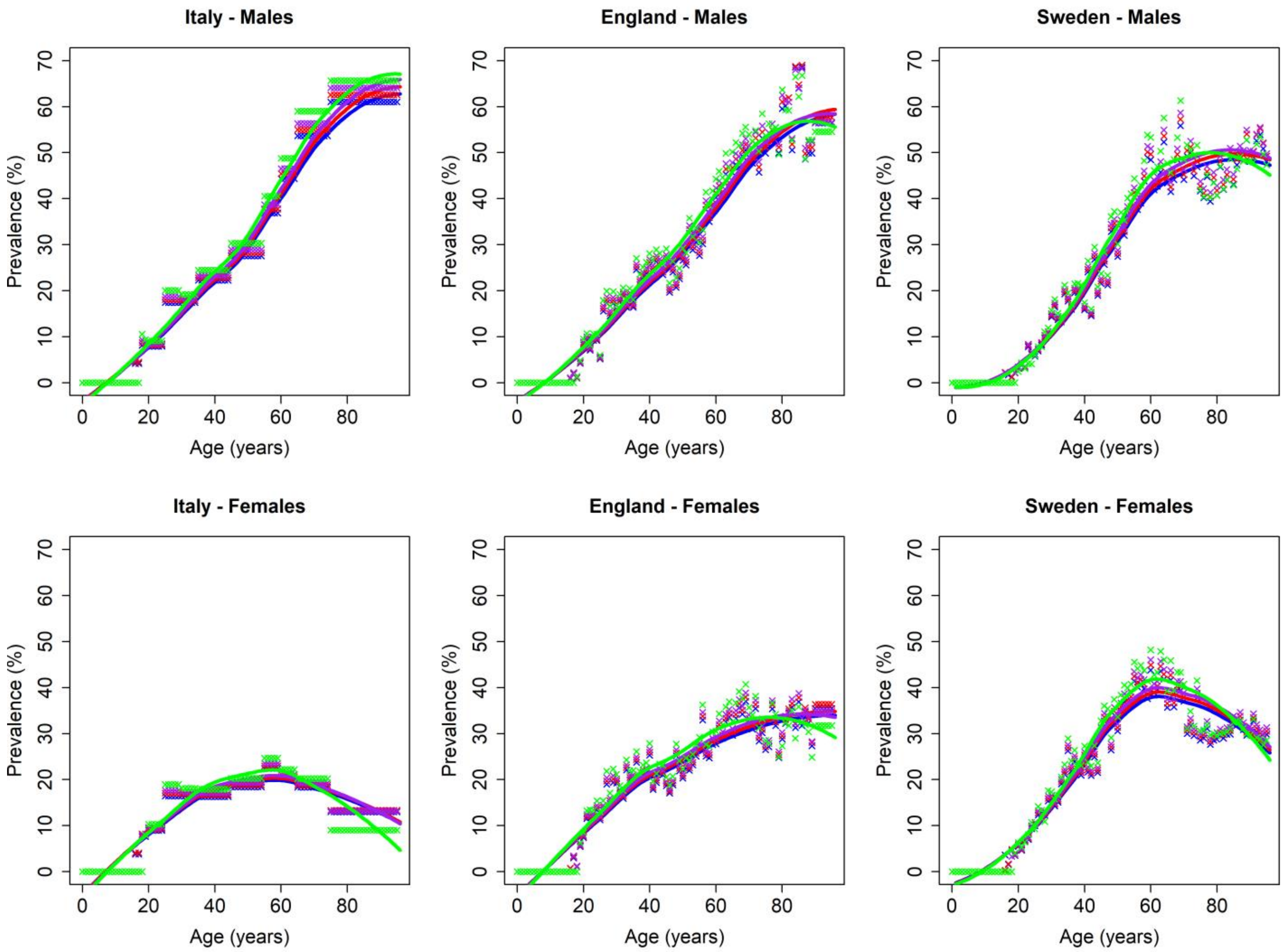

Figure S2: Baseline (2018) **former smoking** prevalences per age, country and intervention (blue: reference scenario; red, purple and green: 5%, 10% and 20% tobacco price increase, respectively). Locally weighted scatterplot smoothing (lowess) was used to create a smooth line through the points. Figure created using the statistical program R (version 3.6.2, www.R-project.org).

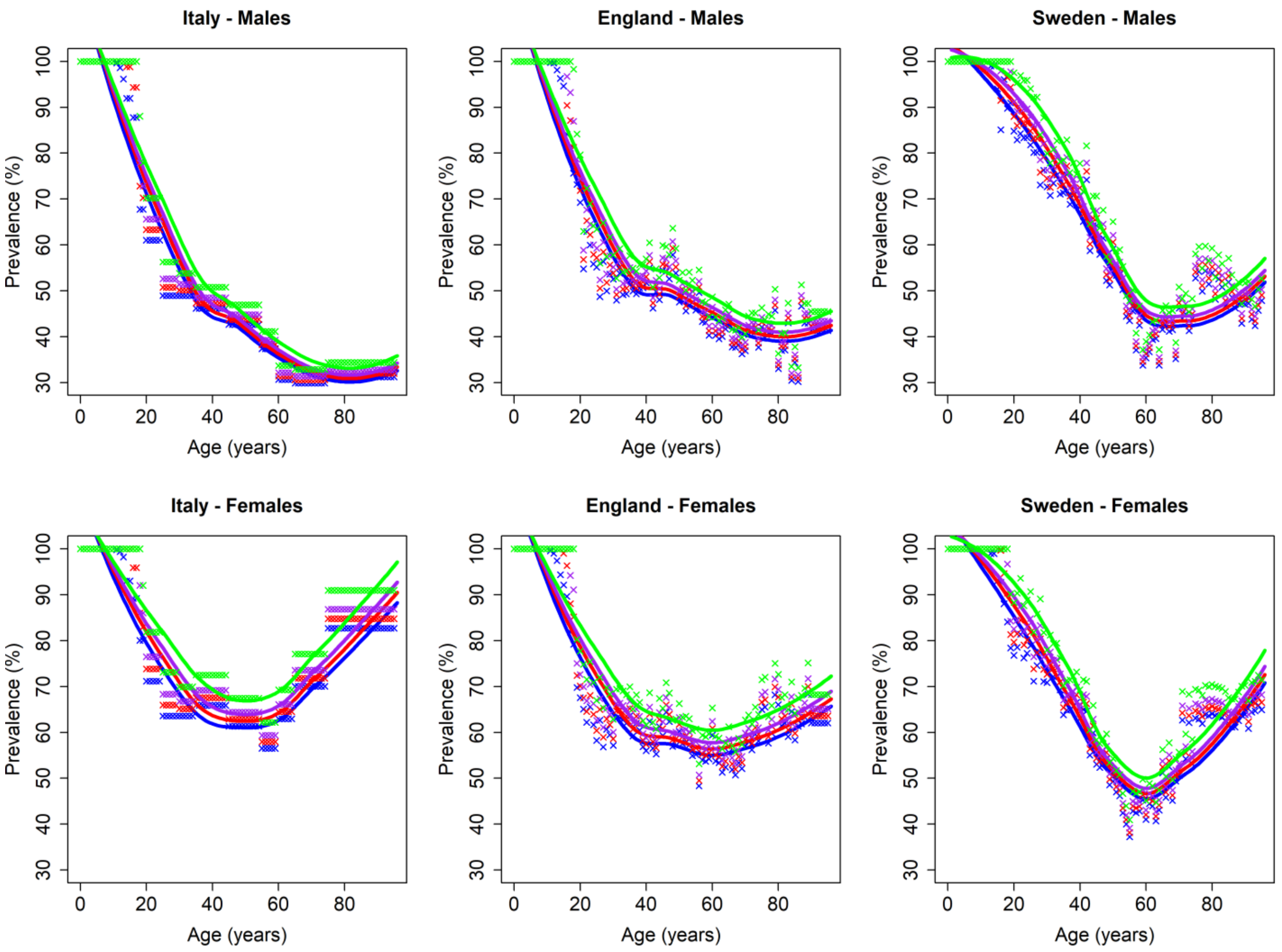

Figure S3: Baseline (2018) **never smoking** prevalences per age, country and intervention (blue: reference scenario; red, purple and green: 5%, 10% and 20% tobacco price increase, respectively). Locally weighted scatterplot smoothing (lowess) was used to create a smooth line through the points. Figure created using the statistical program R (version 3.6.2, www.R-project.org).

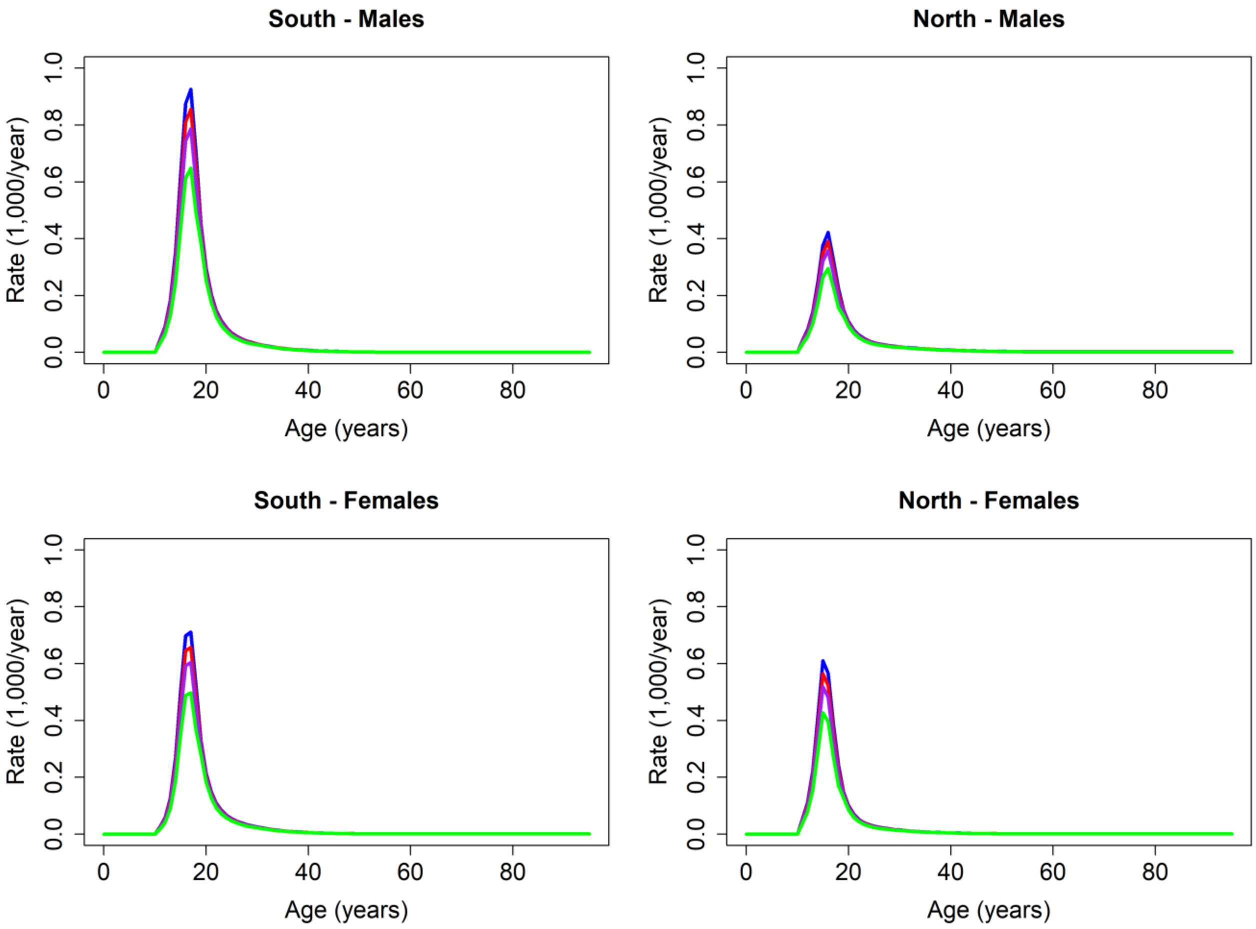

Figure S4: Smoking **initiation** rates per age, region (South for Italy and North for England and Sweden) and intervention (blue: reference scenario; red, purple and green: 5%, 10% and 20% tobacco price increase, respectively). Smoking initiation is only allowed as of 11 years of age. Figure created using the statistical program R (version 3.6.2, www.R-project.org).

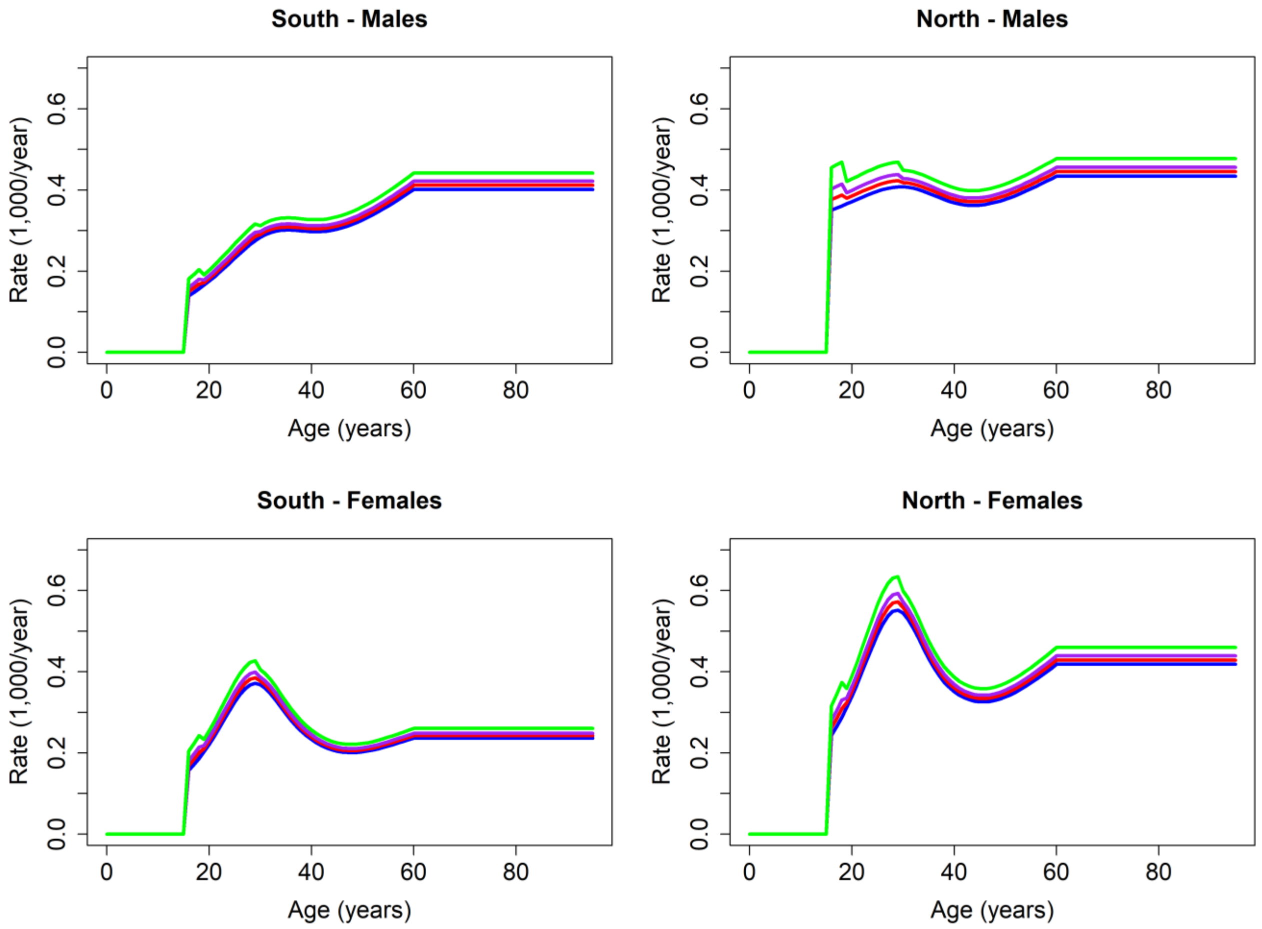

Figure S5: Smoking **cessation** rates per age, region (South for Italy and North for England and Sweden) and intervention (blue: reference scenario; red, purple and green: 5%, 10% and 20% tobacco price increase, respectively). Smoking cessation is only allowed as of 16 years of age. Figure created using the statistical program R (version 3.6.2, www.R-project.org).

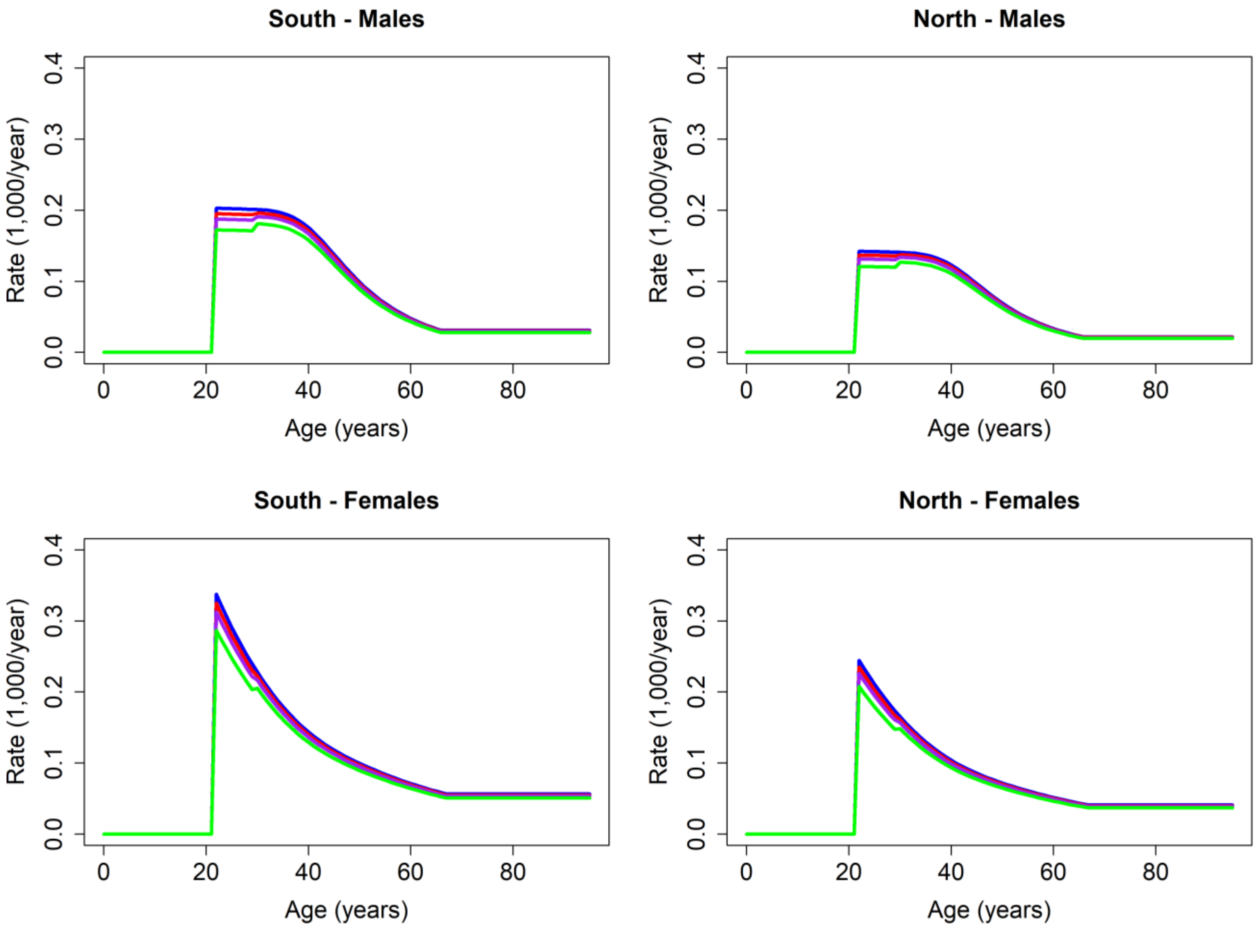

Figure S6: Smoking **restart** rates per age, region and intervention (blue: reference scenario; red, purple and green: 5%, 10% and 20% tobacco price increase, respectively). The same restart rates were used for all countries because sample sizes were insufficient to calculate region-specific restart rates. Smoking restart is only allowed as of 22 years of age. Figure created using the statistical program R (version 3.6.2, www.R-project.org).

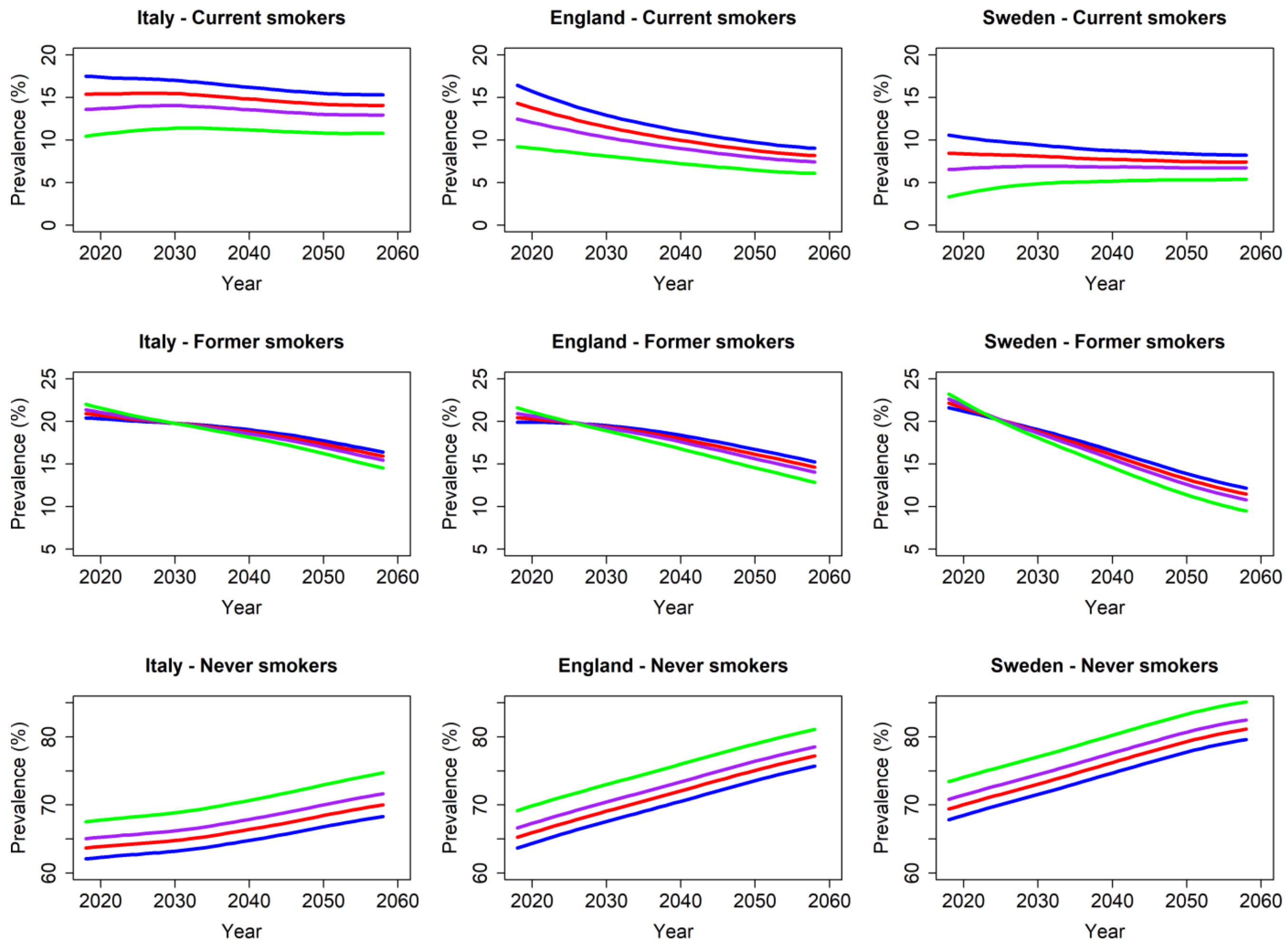

Figure S7: Current, former and never smoking prevalences per country and intervention (blue: reference scenario; red, purple and green: 5%, 10% and 20% tobacco price increase, respectively), projected over a 40-year time period. Note that the range of the y-axes differ per smoking prevalence category. Figure created using the statistical program R (version 3.6.2, www.R-project.org).

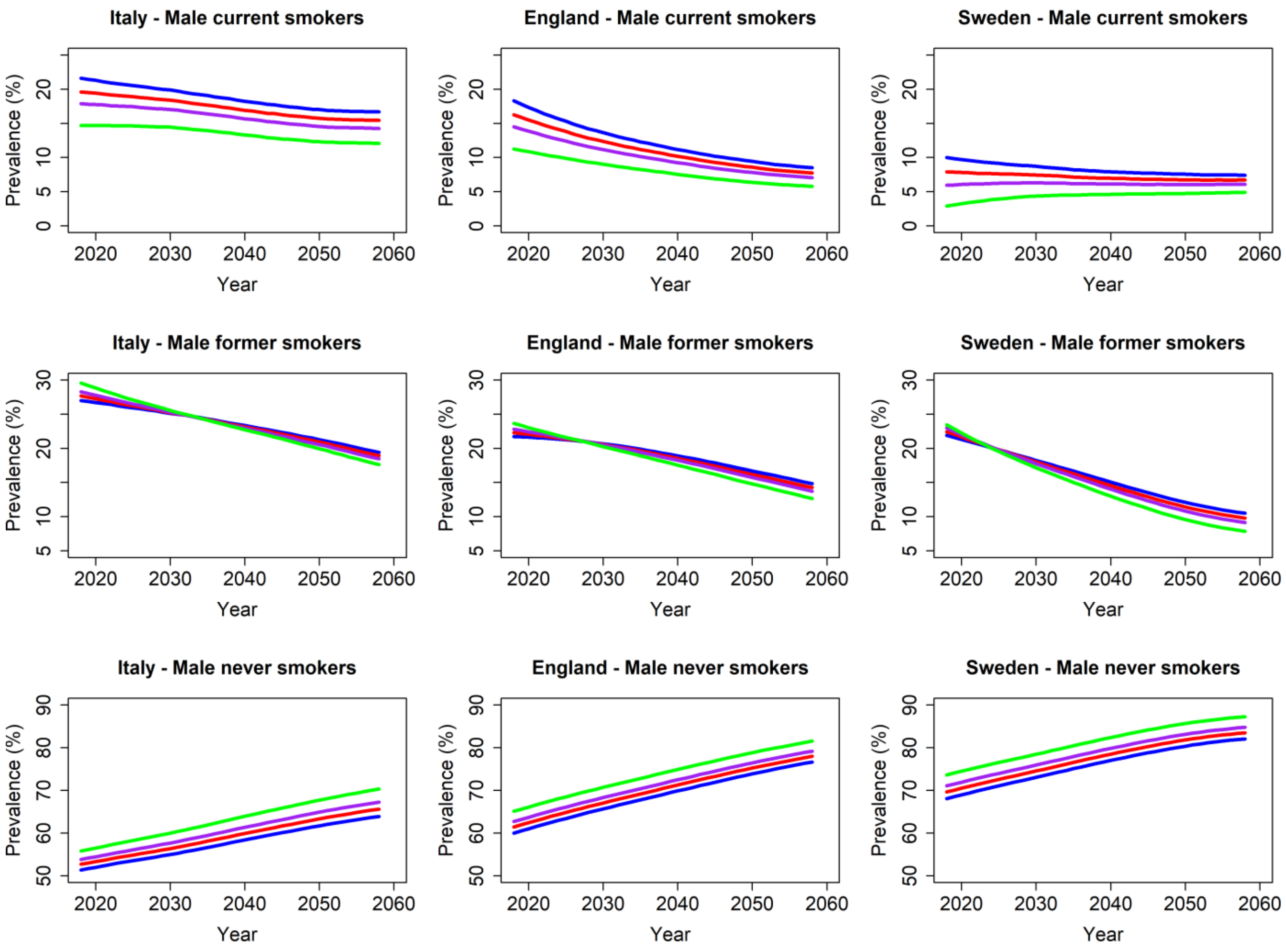

Figure S8: Current, former and never smoking prevalences **among males** per country and intervention (blue: reference scenario; red, purple and green: 5%, 10% and 20% tobacco price increase, respectively), projected over a 40-year time period. Note that the range of the y-axes differ per smoking prevalence category. Figure created using the statistical program R (version 3.6.2, www.R-project.org).

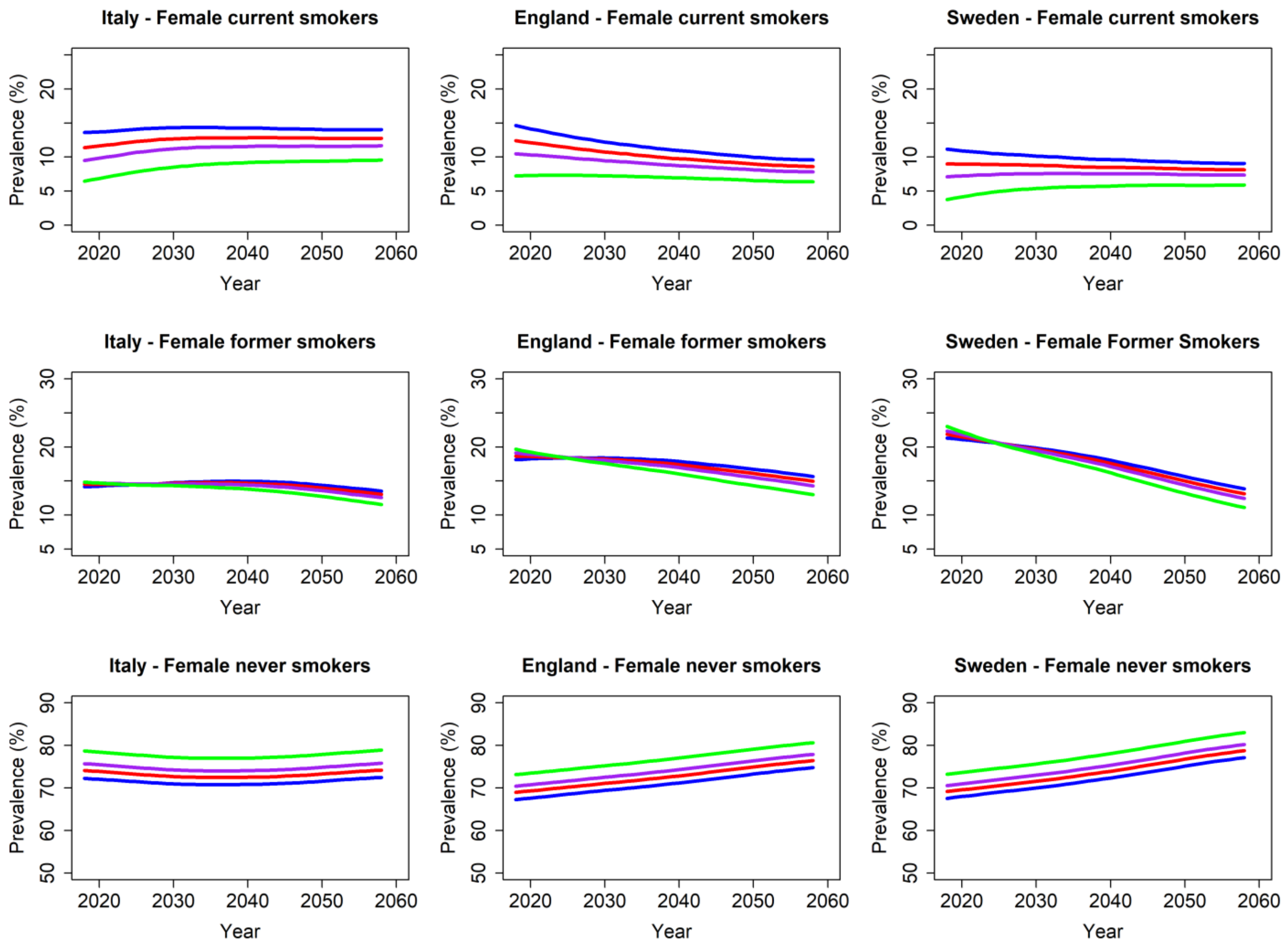

Figure S9: Current, former and never smoking prevalences **<u>among females</u>** per country and intervention (blue: reference scenario; red, purple and green: 5%, 10% and 20% tobacco price increase, respectively), projected over a 40-year time period. Note that the range of the y-axes differ per smoking prevalence category. Figure created using the statistical program R (version 3.6.2, www.R-project.org).

Table S1: COPD prevalence (expressed in %) 10 (2028), 20 (2038) and 40 (2058) years after the implementation of the intervention. Results reported per country, scenario and sex.

		Italy			England			Sweden		
**Sex**	**Scenario**	**2028**	**2038**	**2058**	**2028**	**2038**	**2058**	**2028**	**2038**	**2058**
Men	reference	1.55	1.57	1.27	1.25	1.31	1.17	1.22	1.05	0.78
	5% tobacco price increase	1.52	1.53	1.23	1.22	1.27	1.12	1.19	1.01	0.73
	10% tobacco price increase	1.50	1.48	1.18	1.20	1.22	1.08	1.16	0.97	0.70
	20% tobacco price increase	1.45	1.39	1.09	1.15	1.14	1.00	1.11	0.89	0.63
Women	reference	1.08	1.19	1.02	1.29	1.43	1.28	1.27	1.18	0.86
	5% tobacco price increase	1.06	1.14	0.97	1.25	1.37	1.22	1.25	1.13	0.82
	10% tobacco price increase	1.03	1.09	0.92	1.22	1.32	1.17	1.22	1.09	0.78
	20% tobacco price increase	0.98	1.00	0.83	1.16	1.22	1.08	1.17	1.01	0.70

Table S2: COPD incidence (cases/1,000 person-years) 10 (2028), 20 (2038) and 39 (2057)[a] years after the implementation of the intervention. Results reported per country, scenario and sex.

		Italy			England			Sweden		
**Sex**	**Scenario**	**2028**	**2038**	**2057**	**2028**	**2038**	**2057**	**2028**	**2038**	**2057**
Men	reference	1.99	1.98	1.63	1.68	1.61	1.39	1.53	1.31	0.98
	5% tobacco price increase	1.93	1.91	1.56	1.62	1.55	1.33	1.47	1.24	0.93
	10% tobacco price increase	1.87	1.85	1.49	1.56	1.48	1.28	1.41	1.18	0.88
	20% tobacco price increase	1.76	1.72	1.37	1.44	1.37	1.18	1.29	1.06	0.79
Women	reference	1.07	1.17	1.04	1.56	1.53	1.34	1.27	1.16	0.87
	5% tobacco price increase	1.02	1.11	0.98	1.49	1.46	1.27	1.21	1.10	0.82
	10% tobacco price increase	0.96	1.05	0.92	1.43	1.40	1.21	1.16	1.05	0.78
	20% tobacco price increase	0.87	0.94	0.82	1.31	1.27	1.11	1.06	0.95	0.70

[a] The COPD incidence rate after 39 years (2057) instead of 40 years (2058) is given as DYNAMO does not calculate COPD incidence rates for the last year of the simulation.

Table S3: Price elasticity coefficient sensitivity analysis: **Absolute difference** in overall smoking prevalence, COPD prevalence, cumulative COPD cases saved and gain in overall life-expectancy of a 20 year-old by 2058 as compared to the reference scenario.

		Italy		England		Sweden	
	**Scenario**	**Main analysis[a]**	**Price elasticity sensitivity[b]**	**Main analysis[a]**	**Price elasticity sensitivity[b]**	**Main analysis[a]**	**Price elasticity sensitivity[b]**
Reduction in overall smoking prevalence compared to reference scenario (%)	5% tobacco price increase	-1.2	-0.8	-0.8	-0.5	-0.8	-0.5
	10% tobacco price increase	-2.4	-1.5	-1.6	-0.9	-1.5	-0.9
	20% tobacco price increase	-4.5	-2.8	-2.9	-1.8	-2.8	-1.7
Reduction in COPD prevalence compared to reference scenario (%)	5% tobacco price increase	-0.06	-0.04	-0.05	-0.04	-0.04	-0.04
	10% tobacco price increase	-0.10	-0.09	-0.10	-0.09	-0.08	-0.07
	20% tobacco price increase	-0.19	-0.17	-0.19	-0.17	-0.16	-0.14
Cumulative COPD cases saved compared to reference scenario	5% tobacco price increase	124,364	120,828	125,712	120,783	22,070	21,161
	10% tobacco price increase	247,364	241,142	248,707	240,476	43,654	42,139
	20% tobacco price increase	479,059	467,627	479,302	464,908	83,694	81,424
Gain in overall life expectancy of a male 20-year old compared to reference scenario (years)	5% tobacco price increase	0.10	0.07	0.14	0.11	0.11	0.08
	10% tobacco price increase	0.22	0.16	0.24	0.18	0.20	0.15
	20% tobacco price increase	0.43	0.34	0.45	0.32	0.43	0.30
Gain in overall life expectancy of a female 20-year old compared to reference scenario (years)	5% tobacco price increase	0.10	0.08	0.08	0.06	0.06	0.05
	10% tobacco price increase	0.17	0.13	0.16	0.12	0.11	0.08
	20% tobacco price increase	0.33	0.22	0.31	0.22	0.25	0.17

[a]The price elasticity was -1.5 for < 19 year-olds, -0.75 for 19-29 year-olds, and -0.5 for >29 year-olds; [b]The price elasticity was -0.5 for all ages.

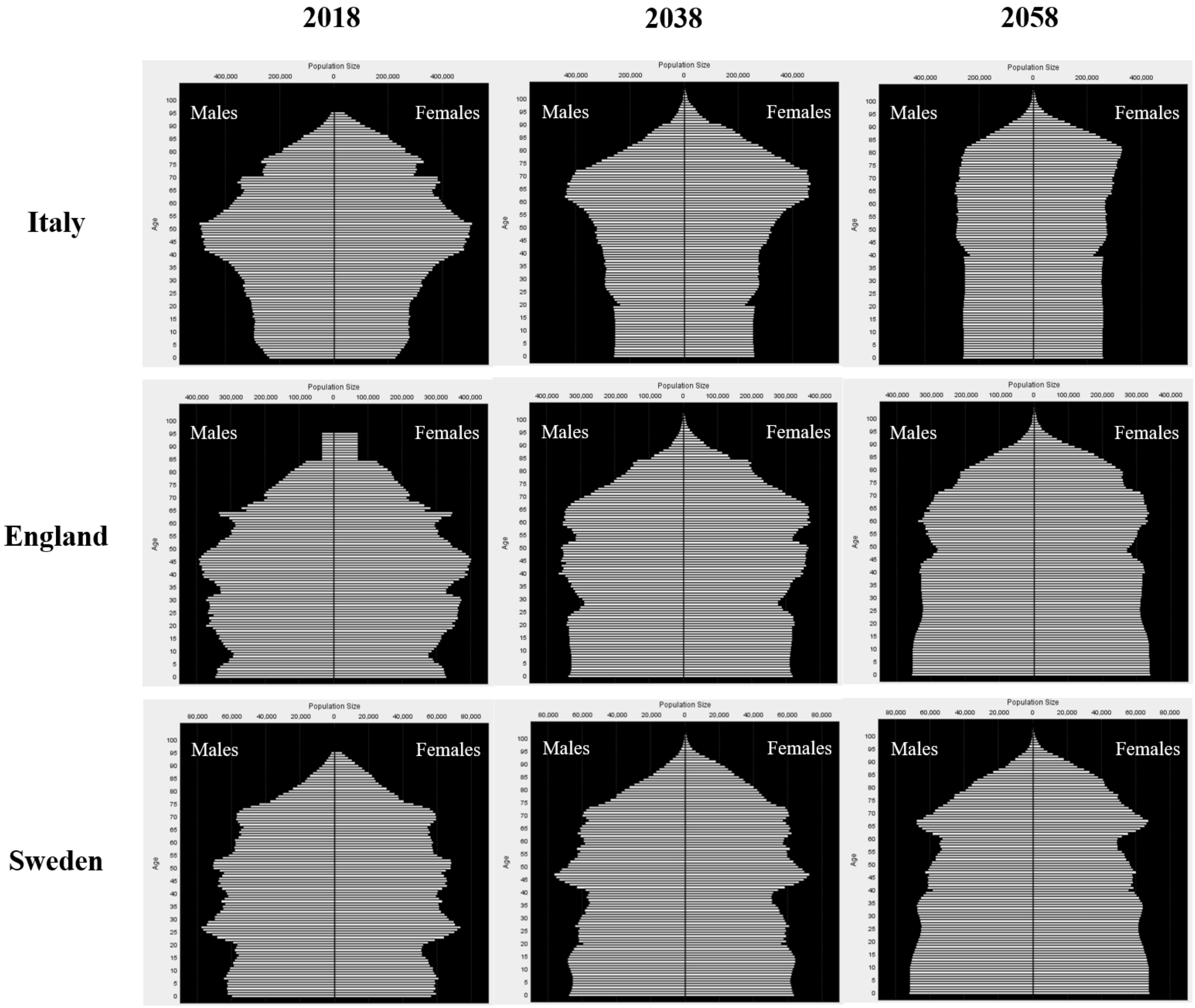

Figure S10: Population structure of Italy, England and Sweden at baseline (2018), as well as projected 20-years (2038) and 40-years (2058) in time. Figure created using the DYNAMO-HIA tool.

Table S4. Secondary analyses **-** COPD burden (prevalence and incidence) 10 (2028), 20 (2038) and 40 (2058) years from the implementation of the intervention. Results reported per country and 30%, 40% and 50% price increase intervention scenarios.

**Health measure**		**Italy**			**England**			**Sweden**		
	**Scenario**	**2028**	**2038**	**2058**	**2028**	**2038**	**2058**	**2028**	**2038**	**2058**
COPD prevalence (%)	reference	1.31	1.37	1.15	1.27	1.37	1.23	1.25	1.11	0.82
	30% tobacco price increase	1.16	1.11	0.87	1.10	1.09	0.96	1.10	0.87	0.62
	40% tobacco price increase	1.12	1.03	0.80	1.06	1.01	0.88	1.06	0.82	0.59
	50% tobacco price increase	1.09	0.96	0.74	1.03	0.95	0.82	1.04	0.79	0.57
COPD incidence **(**cases/1,000 person-years)[a]	reference	1.52	1.57	1.32	1.62	1.57	1.37	1.40	1.23	0.93
	30% tobacco price increase	1.20	1.20	0.99	1.26	1.21	1.05	1.07	0.91	0.69
	40% tobacco price increase	1.11	1.09	0.90	1.17	1.10	0.97	1.00	0.84	0.65
	50% tobacco price increase	1.03	0.99	0.82	1.10	1.02	0.90	0.97	0.81	0.63

[a]The COPD incidence value after 39 years (2057) instead of 40 years (2058) is given as DYNAMO does not calculate COPD incidence rates for the last year of the simulation.

Table S5. Secondary analyses - Absolute gain in overall, COPD -free, and COPD-disability-adjusted cohort life-expectancy (expressed in years) of a 20, 40 and 60 year-old male and female at baseline (2018), as compared to the reference scenario. Results reported per country and 30%, 40% and 50% price increase intervention scenarios.

	**Sex**	**Scenario**	**Italy**			**England**			**Sweden**		
			**Gain in years of life expectancy**			**Gain in years of life expectancy**			**Gain in years of life expectancy**		
			**Overall**	**COPD-free**	**COPD-disability-adjusted**	**Overall**	**COPD-free**	**COPD-disability-adjusted**	**Overall**	**COPD-free**	**COPD-disability-adjusted**
**20-year old**	**Males**	30% tobacco price increase	0.62	0.80	0.53	0.69	0.86	0.57	0.54	0.69	0.45
		40% tobacco price increase	0.80	1.03	0.68	0.89	1.10	0.73	0.56	0.71	0.46
		50% tobacco price increase	0.97	1.24	0.82	1.01	1.25	0.83	0.57	0.73	0.48
	**Females**	30% tobacco price increase	0.53	0.74	0.44	0.47	0.69	0.40	0.36	0.54	0.31
		40% tobacco price increase	0.66	0.91	0.55	0.57	0.83	0.49	0.43	0.64	0.37
		50% tobacco price increase	0.77	1.05	0.63	0.66	0.97	0.57	0.44	0.66	0.38
**40-year old**	**Males**	30% tobacco price increase	0.49	0.65	0.42	0.58	0.78	0.50	0.55	0.75	0.47
		40% tobacco price increase	0.66	0.87	0.56	0.74	0.99	0.63	0.63	0.85	0.54
		50% tobacco price increase	0.79	1.05	0.67	0.90	1.20	0.77	0.70	0.92	0.59
	**Females**	30% tobacco price increase	0.38	0.56	0.33	0.44	0.69	0.39	0.33	0.53	0.30
		40% tobacco price increase	0.50	0.74	0.43	0.61	0.94	0.53	0.38	0.60	0.34
		50% tobacco price increase	0.58	0.84	0.49	0.72	1.11	0.63	0.42	0.66	0.37
**60-year**	**Males**	30% tobacco price increase	0.27	0.36	0.22	0.35	0.47	0.29	0.26	0.38	0.22

**old**		40% tobacco price increase	0.36	0.48	0.30	0.45	0.61	0.37	0.35	0.50	0.30
		50% tobacco price increase	0.45	0.61	0.37	0.49	0.65	0.40	0.39	0.55	0.33
	**Females**	30% tobacco price increase	0.26	0.38	0.22	0.29	0.45	0.25	0.19	0.31	0.16
		40% tobacco price increase	0.35	0.51	0.29	0.33	0.50	0.28	0.23	0.38	0.20
		50% tobacco price increase	0.40	0.56	0.32	0.37	0.55	0.31	0.25	0.41	0.22

Model specification

DYNAMO-HIA simulates an epidemiological causal pathway, where health effects are modelled by linking risk factor exposure to incidence of morbidity and mortality using relative risks. A change in exposure risk factor exposure thus changes disease incidence and in turn disease prevalence and mortality. Total mortality is composed as the mortality due to the disease in question (in this case COPD) and other-cause mortality.

DYNAMO-HIA uses an almost real-life population (stratified by sex and one-year age categories), without consideration for migration. The model is dynamic using one-year time steps for each scenario. Explicit risk-factor states (in this case smoking states) are used so that each simulated individual is classified into a specific risk factor category. Risk factor-specific values are back-calculated using the relative risk from each risk-factor state on disease.

DYNAMO-HIA is a Markov-type model based on a multi-state model. The change of the state depends on current characteristics. The multi-state model is implemented as a partial micro-simulation combining a stochastic micro-simulation to project risk-factor behaviour with a deterministic macro approach for the disease life table. The division into micro- and macro-simulation is done for computation convenience. Both approaches yield the same results[1,2].

The risk-factor part of the model uses micro-simulation. Large numbers of distinctive risk-factor biographies are simulated. Given the age and sex-specific transition probabilities between risk-factor states, which are specified by the user, the risk-factor status of each simulated individual is updated in annual increments.

In the macro module, separate disease life tables are constructed for each risk-factor

biography, which account for competing risks and multiple morbidity. For every risk-factor biography, the probability of disease incidence and mortality over time is calculated, accounting for current age, risk-factor and disease status. Population values are obtained by aggregating the individual biography/diseases life tables.

Table S6: Data inputted into the DYNAMO-HIA model, and their sources.

Data Input		Country	Source
**Population**	Population size	Italy	Italian National Institute of Statistics (ISTAT), resident population at 01/01/2016 (*http://demo.istat.it/pop2016/index.html*)
		England	Office for National Statistics (nomis) - England, census 2011 (*https://www.nomisweb.co.uk/query/construct/summary.asp?mode=construct&version=0&dataset=792*)
		Sweden	Statistics Sweden for the year 2017 (*http://www.statistikdatabasen.scb.se/pxweb/en/ssd/START__BE__BE0101__BE0101A/BefolkningR1860/?rxid=eea59d71-d94f-47c7-936f-2d938d8dae6d*)
	Birth male/female ratio	Italy	Calculated from population size of Italy at age 0 years
		England	Calculated from population size of England at age 0 years
		Sweden	Calculated from population size of Sweden at age 0 years
	Projected number of newborns	Italy	Italian National Institute of Statistics (ISTAT) - 2011-2065, census 2011
		England	Office for National Statistics (nomis) - England (*https://www.nomisweb.co.uk/query/construct/summary.asp?mode=construct&version=0&dataset=2009*)
		Sweden	Statistics Sweden (*http://www.scb.se/en/finding-statistics/statistics-by-subject-area/population/population-projections/population-projections/pong/tables-and-graphs/the-future-population-of-sweden-20162060/children-per-woman-and-forecast/*)
	Overall mortality	Italy	DYNAMO-HIA; country-specific estimates for Italy[a]
		England	DYNAMO-HIA; country-specific estimates for the UK[a]
		Sweden	DYNAMO-HIA; country-specific estimates for Sweden[a]
	Overall disability	Italy	DYNAMO-HIA; country-specific estimates for Italy[a]
		England	DYNAMO-HIA; country-specific estimates for UK[a]
		Sweden	DYNAMO-HIA; country-specific estimates for Sweden[a]

**Risk Factor**	Smoking prevalence	Italy	Age <14 years: ECRHS 1 (Italian centres only) and GEIRD observational studies<br>Age $\geq$14 years: Italian National Institute of Statistics (ISTAT), annual multi-purpose survey 2015
		England	Health Survey for England (2010-2014)[b]
		Sweden	Age <16 years: Health Behaviour in School-Aged Children, 2012/2013 (Swedish version)<br>Age $\geq$16 years: GALEN observational study
	Duration (years) smoked by ex-smokers	Italy	DYNAMO-HIA; country-specific estimates for Italy[a]
		England	DYNAMO-HIA; country-specific estimates for UK[a]
		Sweden	DYNAMO-HIA; NB, data calculated for UK are also used for Sweden[a]
	Smoking initiation, restart and cessation probabilities	Italy	Pooled data from several large observational studies in Southern Europe (Italy, Portugal, Spain)[3,4]
		England and Sweden	Pooled data from several large observational studies in Northern Europe (Denmark, Finland, Iceland, Norway, Sweden, UK)[3,4]
	Relative risk smoking on total mortality	All	The same estimates provided within DYNAMO-HIA used for all countries.<br>$\geq$ 35 years: 2.07 (males), 1.74 (females)
**Disease**	COPD excess mortality	Italy	DYNAMO-HIA; country-specific estimates for Italy[a]
		England	DYNAMO-HIA; country-specific estimates for the UK[a]
		Sweden	DYNAMO-HIA; country-specific estimates for Sweden[a]
	COPD incidence	Italy	DYNAMO-HIA; country-specific estimates for Italy[a]
		England	DYNAMO-HIA; country-specific estimates for the UK[a]
		Sweden	DYNAMO-HIA; country-specific estimates for Sweden[a]
	COPD prevalence	Italy	DYNAMO-HIA; country-specific estimates for Italy[a]
		England	DYNAMO-HIA; country-specific estimates for the UK[a]
		Sweden	DYNAMO-HIA; country-specific estimates for Sweden[a]

Relative risk of smoking on COPD	All	The estimates provided within DYNAMO-HIA used for all countries[a]<br>50-54 years: 8.13 (males), 12.92 (females); 55-59 years: 9.80 (males), 9.47 (females); 60-64 years: 13.21 (males), 11.19 (females); > 65 years: 19.83 (males), 14.72 (females)

[a]Freely available from www.dynamo-hia.eu/en/reference-data; [b]Data freely available from beta.ukdataservice.ac.uk and can be downloaded after user registration.

Description of smoking prevalence calculations per country

Age-specific smoking prevalences at young ages are generally not available from official sources. For Italy, smoking prevalences among individuals aged 11-13 years were retrospectively calculated using pooled observational data on self-reported age of smoking initiation, using data from three Italian centres in the European Community Respiratory Health Survey (ECRHS) I (501 males and 520 females)[5] and from the Italian Gene- Environment Interactions in Respiratory Diseases (GEIRD) study (6,470 males and 6,895 females)[6]. For those aged 14-96, smoking prevalences were available from the 2015 Italian National Institute of Statistics (ISTAT) annual multi-purpose survey (www.demo.istat.it/pop2016/index.html).

For England, data from the Health Survey for England (https://digital.nhs.uk/data-and-information/areas-of-interest/public-health/health-survey-for-england-health-social-care-and-lifestyles) were used to generate smoking prevalences. To increase the stability of the estimates, data from the latest available five-year period (2010-2014) were combined. For individuals aged 16-95, smoking prevalences were directly available from this source. For those aged 11-15, smoking prevalences were retrospectively calculated per year of birth using self-reported age of smoking initiation. For these younger ages only, we took the average prevalences across those born between 1992-1996 in the most recent available five-year period, to minimize potential cohort effects.

For Sweden, smoking prevalences for individuals aged 11-15 were obtained from the World Health Organization report on Health Behaviour in School Aged Children study conducted in 2012/2013 and were based on reports of smoking at least once a week[7]. For those aged 16-75, smoking prevalences were calculated from the Swedish centers participating in the Global Allergy and Asthma European Network (GA2LEN) (N=26,648, www.ga2len.net/index.html). For individuals 76-95, we used the yearly percent change observed in England in the same ages and applied it to the Swedish data, as no appropriate local source could be identified.

REFERENCES

1. Van Imhoff, E. & Post, W. Microsimulation methods for population projection. *Population* **10**, 97-138 (1998).

2. Karon, J. Alternative decision modelling techniques for the evaluation of health care technologies: Markov processes verses discrete event simulation. *Health Econ.* **12**, 837-848 (2003).

3. Marcon, A. *et al.* Trends in smoking initiation in Europe over 40 years: A retrospective cohort study. *PLOS ONE* **13**, e0201881 (2018).

4. Pesce, G. *et al.* Time and age trends in smoking cessation in Europe. *PLOS ONE* **14**, e0211976 (2019).

5. Burney, P. G., Luczynska, C., Chinn, S. & Jarvis, D. The European Community Respiratory Health Survey. *Eur. Respir. J.* **7**, 954–960 (1994).

6. de Marco, R. *et al*. The Gene-Environment Interactions in Respiratory Diseases (GEIRD) Project. *Int. Arch. Allergy Immunol.* **152**, 255–263 (2010).

7. World Health Organization. Growing up unequal: gender and socioeconomic differences in young people's health and well-being. Health Behaviour in School-Aged Children (HBSC) Study (2013/2014 survey, 150-152). http://www.euro.who.int/en/publications/abstracts/growing-up-unequal.-hbsc-2016-study-20132014-survey (2016).
